# In utero myelomeningocele repair and high-risk bladder pattern. a prospective study

**DOI:** 10.1590/S1677-5538.IBJU.2022.0053

**Published:** 2022-03-30

**Authors:** Antonio Macedo, Sérgio Leite Ottoni, Antonio Moron, Sergio Cavalheiro, Marcela Leal da Cruz

**Affiliations:** 1 Departamento de Pediatria Universidade Federal de São Paulo São Paulo SP Brasil Departamento de Pediatria, Universidade Federal de São Paulo - Unifesp, São Paulo, SP, Brasil;; 2 Departamento de Urologia CACAU-NUPEP São Paulo SP Brasil Departamento de Urologia, CACAU-NUPEP, São Paulo, SP, Brasil;; 3 Departamento de Obstetrícia-Medicina Fetal Universidade Federal de São Paulo São Paulo SP Brasil Departamento de Obstetrícia-Medicina Fetal, Universidade Federal de São Paulo - Unifesp, São Paulo, SP, Brasil;; 4 Hospital Maternidade Santa Joana São Paulo SP Brasil Hospital Maternidade Santa Joana, São Paulo, SP, Brasil;; 5 Departamento de Neurocirurgia Universidade Federal de São Paulo São Paulo SP Brasil Departamento de Neurocirurgia, Universidade Federal de São Paulo - Unifesp, São Paulo, SP, Brasil

**Keywords:** Meningomyelocele, Urinary Bladder, Urinary Bladder, Neurogenic

## Abstract

**Objectives:**

High-risk bladder pattern can be defined by Urodynamic Evaluation (UE) as overactive bladder with detrusor leak point pressure higher than 40 cmH2O and/or higher filling pressures also above 40 cmH2O. We wanted to evaluate response to treatment in myelomeningocele patients operated *in utero* in this subgroup.

**Patients and Methods:**

From our prospective cohort of *in utero* MMC we have identified patients in the high-risk group. Treatment consisted of anticholinergics (Oxybutynin 0.2 mg/Kg) 2 or 3 times daily in association with CIC. At every UE, patients were reclassified in high-risk or low-risk patterns. Patients not responding were proposed bladder reconstruction or diversion according to age.

**Results:**

Between 2011 to 2020, we have been following 121 patients and 60 (49.6%) of them were initially categorized as high-risk. The initial UE was performed at a mean age of 7.9 months and detrusor overactivity was found in 83.3% (mean maximum pressure of 76.5cmH20). When evaluating patients with 2 or more UE, we identified 44 patients (follow-up: 36.8months). It was observed in the group of patients who underwent 2 to 5 UE, that response to treatment was validated by the finding of 40% of low-risk bladder patterns in the second UE and between 62% to 64% in the third to the fifth UE. The incidence of surgery was 13.3%.

**Conclusions:**

Early urological treatment of high-risk bladder pattern was effective in approximately 60%. We reinforce the need to correctly treat every patient with myelomeningocele, in accordance with UE, whether undergoing *in utero* or postnatal treatment.

## INTRODUCTION

*In utero* myelomeningocele (MMC) repair has shown benefits with reduced need for ventriculoperitoneal shunt and improved motor status according to data published in 2011 in MOMS (Management of Mylomeningocele Study) ( 1 ). The potential improvement in the bladder function is a controversial subject, as the different studies published in the post-MOMS era are conflicting. When analyzing prospective studies, the American groups involved in MOMS ( 2 , 3 ) and the Zurich group ( 4 , 5 ) suggest benefits of *in utero* MMC to the bladder function, while our studies in São Paulo go in the opposite direction and shows no improvements to bladder function, regardless if MMC closure had been *in utero* or postnatal ( 6 - 12 ).

The knowledge about myelomeningocele and urological presentation and outcome in ongoing advocates early clean intermittent catheterization (CIC) and antimuscarinic drugs should be indicated upon results of urodynamic studies. ( 13 ) There are still no serum indicators that allow recording renal damage before the lesion established on DMSA scintigraphy ( 14 ). Reconstructive surgery (such as enterocystoplasty) was indicated for patients who were refractory to clinical treatment. For those who were at an early age, vesicostomy should be regarded as an alternative in order to postpone definitive surgery ( 15 ).

Our cohort is characterized by a prospective follow-up of patients who underwent *in utero* surgery as of 2011. We propose urological treatment based on initial categorization of four main patterns of bladder behavior at first presentation and subsequently after each appointment. ( 7 )

Most studies assessing response to treatment of high-risk bladder patterns in myelomeningocele are retrospective and belong to the postnatal MMC repair aera. To our knowledge, this is the first paper to study specifically the outcome of patients operated *in utero* treated with anticholinergics and CIC only in a prospective course and this is the rationale of our manuscript.

The aim of this study after selecting the subgroup of patients categorized as high-risk pattern (overactive bladder with detrusor leak point pressure higher than 40 cmH2O and higher filling pressures also above 40 cmH2O) was to define the rate of response to treatment and clinical evolution after initiation of anticholinergics and CIC.

## PATIENTS AND METHODS

In 2011 we started a prospective urological follow-up protocol of patients with MMC operated in utero. This protocol received approval from our Ethic Committee and IRB 34234. This study was based on a prospective protocol to categorize and treat patients with the same procedures and retrospective analysis of patients’ files searching outcomes according to the aim of the study. After clinical evaluation and radiological exams: urinary tract ultrasound (US), voiding cystourethrography (VCUG) and urodynamic evaluation (UE), patients were categorized^7^ and treated as follows: 1) High-Risk Pattern (overactive pattern with detrusor leak point pressure higher than 40 cm H2O and/or higher filling pressures above 40 cm H2O in the absence of a detrusor contraction) anticholinergics (oxybutynin 0.2 mg/Kg) 2 or 3 times daily in association with CIC every 4 hours, 5 times a day, 2) Incontinent Pattern (overactive bladder with detrusor leak point pressure lower than 40 cm H2O or stable bladder but leaking below 40 cm H2O) and Normal Pattern (stable bladder cystometry without leakage) - only clinical surveillance, and 3) Underactivity Pattern (underactive bladder with post-void residual urine) - Only CIC. Our protocol suggests assessments at 6-month intervals until stability of the urodynamic pattern and then yearly controls with further US and UE. All urodynamic evaluations were performed using the same device and by the same investigators. We estimate bladder capacity according to the Holmdahl formula, bladder capacity in mL = 38 + 2.5 x age in months ( 16 ).

The high-risk group is known to be that one with higher risk to the upper urinary tract and the cutoff of 40 cm H20 of detrusor pressure is well recognized in the literature. Thus, we retrospectively reviewed our prospectively fed database. All clinical information, imaging exam results, response to initial treatment and serial urodynamic evolution were collected. At every urodynamic evaluation in the clinical follow-up, patients were reclassified at high-risk or low-risk risk pattern, if the findings of overactive bladder with detrusor leak point pressure higher than 40 cmH2O and higher filling pressures also above 40 cm H2O were normalized. Thus, it was possible to serially define the percentage of response to the proposed treatment. Another interesting aspect was to be able to define the incidence of lower urinary tract surgery in this high-risk group.

## RESULTS

Within the interval from 2011 to December 2020, our cohort was formed by 121 patients, and of these, 60 were categorized at high-risk (49.6%) and included in this study. The mean time of follow-up was 27.9 months, and the median was 22 months (ranging from 1 to 91 months). The mean gestational age at diagnosis was 21.1 weeks and mean age at *in utero* surgery was 25.7 weeks. The mean age at birth was 31.1 weeks. The incidence of ventriculoperitoneal shunts was 12.1% (n=7). The mean age of the first urological evaluation was 6.9 months (median 5 months). The initial ultrasound was performed with a mean of 7.31 months (median 5 months) in 58 patients. Hydronephrosis was observed in 27.6% (n=16) and thick-walled bladder in 34.5%.

The VCUG was performed in 58 patients at a mean age of 9 months (median 6 months), showing irregularly shaped bladder in 39.7%, dilatation of the urethra in 36.8%, suggesting vesico-sphincter dyssynergia in 36.8%. The diagnosis of vesicoureteral reflux was made in 27.6% of the cases (n=16), being bilateral in 10.3% (n=6). The grade distribution per renal units (RU) was: GIII: 3, GIV: 11, and GV: 7 RU.

The initial UE was performed at a mean age of 7.9 months (median 5 months) and showed hyperactivity in 83.3%, with mean maximum pressure of 76.5 cm H20 (median 72.5 cm H20). Bladder compliance was normal in 13.6%, decreased in 61%, and could not be determined in 25.4% due to leakage. Similarly, bladder capacity was normal in 37.3%, decreased in 57.6% and could not be determined in 5.1% due to leakage.

When evaluating patients with 2 or more UE, we identified 44 patients, who were followed for a mean follow-up period of 36.8 months (median 28.5 months). Demographic data of this group is presented in Table-1 . This subgroup allowed us to evaluate the response to treatment according to objective parameters and mainly based on the UE. We observed episodes of urinary tract infection (UTI) in 28 patients (63.6%), with a mean of 1.9 episodes per patient. The need for hospitalization to treat UTI was 25%. We define UTI as a febrile event with abnormal urinalysis and positive cultures yielding more than 100,000 colonies per mL.

**Table 1:** Demographic data.

Demographic Data		
**n**	44	
**Sex**	Female	Male
	21 (47.7%)	23 (52.3%)
**Age at first presentation**	Average	Median
	6.8 months	5.0 months
**Age at last appointment**	Average	Median
	45.3 months	40 months
**Response to clinical treatment** **(at last urodynamic evaluation)**	High-risk	Low-risk
	24 (54.5%)	20 (45.5%)

The second UE was performed at a mean age of 18.8 months (median 17.5 months) and showed overactive detrusor contractions in 32.6% (n=14), mean maximum pressure of 49.1 cmH2O (median 50 cmH2O), normal bladder compliance in 54.4%, decreased compliance in 40.9% and could not be determined due to leakage in 4.5%. The recategorization of the bladder pattern by the classification of Leal da Cruz et al. ( 7 ) was high-risk in 61.4% (n=27) and low-risk in 38.6% (n=17) in the second exam.

**Figure 1:**
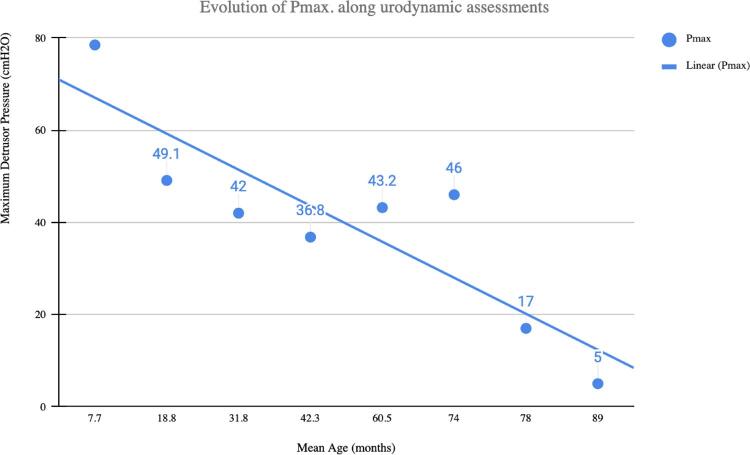
Evolution of the maximum detrusor pressure throughout the urodynamic evaluations registered during the follow-up period.

Figure-1 shows the evolution of the maximum pressure obtained in the subsequent urodynamic evaluations registered during the follow-up period. Maximum detrusor pressure was the main parameter to recategorize patients at each follow-up.

**Figure 2:**
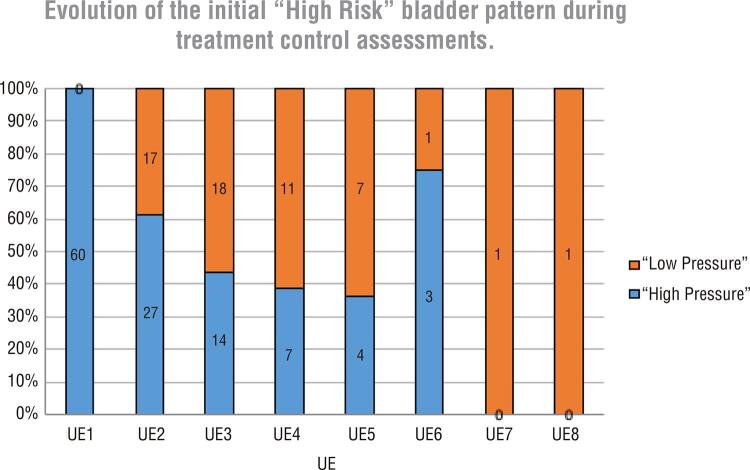
Number of patients by sequence of urodynamic evaluations (EU) performed UE1 (n:60) UE2 (n:44), UE3 (n:32), UE4 (n:18), UE5 (n:11), UE6 (n:4), UE7 (n:1), UE8 (n:1) and percentage of high risk and low risk bladder patterns.

The records of serial urodynamic evaluations allow us to categorize the bladder pattern at every exam, and Figure-2 shows the percentage of high-risk and low-risk patterns according to the number of UE performed. It is observed that patients who underwent 3, 4 and 5 urodynamic evaluations had a response close to 60% of change in the bladder pattern to low-risk.

The precise values of the mean maximum pressure in the high and low-risk subgroups according to data from the second to the fifth urodynamic evaluations can be seen in Table-2 .

**Table 2:** Mean maximum pressure in the high and low-risk subgroups according to the second to the fifth urodynamic evaluations.

Bladder Pattern	Pmax. UED2 (cmH2O)	Pmax. UED3 (cmH2O)	Pmax. UED4 (cmH2O)	Pmax. UED5 (cmH2O)
Low-risk	16.76	18.78	16.91	17.14
High-risk	69.52	71.93	68	88.75

In this series, the following procedures were performed: vesicostomy (n: 3), surgery for treatment of vesicoureteral reflux (n: 2. Note: 1 patient with 2 surgeries), and bladder augmentation (3, one patient with associated Macedo-Malone), which signified a surgery incidence of 13.3%. The decision between bladder reconstruction (augmentation) or diversion (vesicostomy) has been taken according to age with 3 years and half as a cutoff.

## DISCUSSION

The proactive management of children with spina bifida is based on early bladder pattern categorization. We have followed the same urological protocol for neuropathic bladders since 1999 and this has enabled us to compare the clinical presentation of patients with myelomeningoceles operated *in utero* and postnatally ( 10 ). We were not able to follow high-risk bladder patterns simultaneously for postnatal MMC repair because in our Institution all cases are referred for *in utero* repair since 2011. We are aware that the progression of kidney failure towards end-stage renal disease can be reduced if adequately treated.

Although there is preliminary literature available comparing initial bladder patterns after *in utero* MMC repair, there is little information about the clinical outcome after treatment for this new subgroup of patients.

The MOMS urological follow-up study showed that 24% in the prenatal group vs 4% in the postnatal group (RR 5.8, 95% CI 1.8-18.7) were reported to be voiding volitionally ( 3 ). Augmentation cystoplasty, vesicostomy and urethral dilation did not differ between the two groups. Authors reported that in spite that most patients were in diapers or in a CIC regimen, there was a trend for higher volitionally voiding in the *in utero* operated patients. Interestingly, the conclusion of the authors was that *in utero* closure should not be performed solely based on urological outcome ( 3 ). One aspect that is not clear in the manuscript was that this beneficial conclusion came out from a non-reported number of cases by parental information through phone interviews only and not confirmed by medical analysis. The meaning of volitional voiding by neuropathic patients is accepted only if no residual volumes are found and the authors did not respond on that. Another limiting factor of this paper was the presence of multiple clinical providers during follow-up, not necessarily having the same protocol in their counseling.

The Zurich group recently presented their data for *in utero* MMC repair and urological outcome. They performed UDS at 2 weeks, 6, 12, 18 and 24 months, followed by yearly control and included 82 patients. The last UDS was normal in 25 patients (32%), in contrast to 66% (54/82) in the newborn period. Only 35 patients had a 3 year follow-up showing normal bladder parameters in 6 cases (17%) ( 5 ). Their most recent findings are in correspondence with our results that suggest no protective effect of fetal myelomeningocele surgery towards the lower urinary tract. One strong aspect of this paper is that, similarly to our data, these authors present a very homogeneous follow-up protocol with validated UE.

We have recently reported on our experience in patients operated *in utero* and presenting with sphincter insufficiency patterns. We were able to follow 30 patients in a database of 117 patients operated *in utero* categorized in the incontinent pattern (leaking at lower pressure: < 40cm H20). From those, 23 had repeated UDS available to record clinical outcome with a follow-up of 24.5 months (median: 15 months). We observed that no change in the pattern was found in 43.47% and for those leaking at a lower pressure (<30cm H20), we could predict maintenance of the incontinent pattern in 70% ( 12 ).

Other authors have performed similar studies after post-natal MMC repair. Sager et al. did a retrospective analysis after studying 60 cases of MMC presenting at an age below 1 year. They observed the incidence of high-risk bladder patterns in 50% of their population and 30% had a diagnosis of detrusor-sphincter dyssynergia ( 17 ). This data is very similar to our findings. In a study performed with the first 100 patients of prospective follow-up, of which 95 underwent urodynamic evaluation, we found that the high-risk group represented 52.6% ( 10 ).

We wanted to estimate in this prospective analysis, the response to treatment and the incidence of surgery, which indirectly represents failure of conservative treatment. Importantly, in this group we reviewed 172 urodynamic evaluations only for the high-risk group, which gave us a mean of 3.9 evaluations per patient. Noteworthy, all evaluations were performed in the same device and by the same investigator (AMJ), which provides homogeneity to the UE data never seen in neurological patients. At our service, a urodynamic exam is performed at the office and together with the medical visit, with the assistance of an urotherapist nurse. The immediate analysis of the results by the attending physician allows for decision-making during the medical appointment.

An interesting characteristic of our service is also the adherence of patients from several localities in the country, who return on a yearly basis for control with the neurosurgeon (SC), pediatric orthopedics and pediatric urologist (AMJ). As additional data, the geographic origin of the 60 patients initially enrolled in this study is observed: North: 5%; Northeast: 8,3%; Midwest: 10%; South: 6.7%; Southeast: 68.3%; and another country: 1.7%.

The UE findings that allow classifying the bladder pattern as high-risk and low-risk are shown in Graph 2. It was observed, mainly in the group of patients who underwent 2 to 5 UE, that the presumable response to treatment could be validated by the finding of 40% of low-risk bladder patterns in the second UE and between 62% to 64% in the third to the fifth UE.

The incidence of surgery is ultimately considered a failure of conservative treatment (anticholinergics and CIC). In this series, the following procedures were performed: vesicostomy (n: 3), surgery for treatment of vesicoureteral reflux (n: 2. Note: 1 patient with 2 surgeries), and bladder enlargement (3, one patient with associated Macedo-Malone), which signified a surgery incidence of 13.3%.

A limitation of our study was that a small number of patients have abandoned the treatment in our Institution. On the other hand, it did not affect our conclusions, as far as only the interval between first and last appointment with us have been counted for our mean follow-up in the study.

## CONCLUSIONS

Thus, we can conclude that early urological treatment using anticholinergics and CIC of patients with myelomeningocele and who initially presented with a high-risk bladder pattern was effective in approximately 60% of the cases. The incidence of surgery was 13.3% in this group, with a mean follow-up of 36.8 months (median 28.5 months). Therefore, we reinforce the need to correctly treat every patient with myelomeningocele, in accordance with objective parameters and based on urodynamic evaluation, whether undergoing *in utero* or postnatal treatment.
